# Combining Immunotherapy with Oncogene-Targeted Therapy: A New Road for Melanoma Treatment

**DOI:** 10.3389/fimmu.2015.00046

**Published:** 2015-02-09

**Authors:** Mariana Aris, María Marcela Barrio

**Affiliations:** ^1^Centro de Investigaciones Oncológicas-Fundación Cáncer, Buenos Aires, Argentina

**Keywords:** melanoma, oncogene-targeted therapy, immunotherapy, combination therapy

## Abstract

Cutaneous melanoma arises from the malignant transformation of skin melanocytes; its incidence and mortality have been increasing steadily over the last 50 years, now representing 3% of total tumors. Once melanoma metastasizes, prognosis is somber and therapeutic options are limited. However, the discovery of prevalent BRAF mutations in at least 50% of melanoma tumors led to development of BRAF-inhibitors, and other drugs targeting the MAPK pathway including MEK-inhibitors, are changing this reality. These recently approved treatments for metastatic melanoma have made a significant impact on patient survival; though the results are shadowed by the appearance of drug-resistance. Combination therapies provide a rational strategy to potentiate efficacy and potentially overcome resistance. Undoubtedly, the last decade has also born a renaissance of immunotherapy, and encouraging advances in metastatic melanoma treatment are illuminating the road. Immune checkpoint blockades, such as CTLA-4 antagonist-antibodies, and multiple cancer vaccines are now invaluable arms of anti-tumor therapy. Recent work has brought to light the delicate relationship between tumor biology and the immune system. Host immunity contributes to the anti-tumor activity of oncogene-targeted inhibitors within a complex network of cytokines and chemokines. Therefore, combining immunotherapy with oncogene-targeted drugs may be the key to melanoma control. Here, we review ongoing clinical studies of combination therapies using both oncogene inhibitors and immunotherapeutic strategies in melanoma patients. We will revisit the preclinical evidence that tested sequential and concurrent schemes in suitable animal models and formed the basis for the current trials. Finally, we will discuss potential future directions of the field.

## Introduction

The American Cancer Society projected 76,100 new cases of melanoma in the United States in 2014 (1). These cases represent <2% of total skin cancer diagnoses, but account for an overwhelming proportion of skin cancer deaths. Melanoma incidence has been increasing for at least 30 years, and between 2006 and 2010 the incidence rate among Caucasians increased by 2.7% per year. Though incidence is rising, mortality has been declining rapidly in Caucasians under 50: from 2006 to 2010, mortality rates decreased by 2.6% per year in men and by 2.0% per year in women. In contrast, among Caucasians 50 and older, mortality increased by 1.1% per year in men and by 0.2% per year in women during this same time period. Even with this decline in mortality, the American Cancer Society predicted 9,710 deaths from melanoma in 2014 (1).

Standard cutaneous melanoma (CM) treatment consists of surgical removal of the primary tumor and surrounding normal tissue; a sentinel lymph node is routinely biopsied to determine stage (2). More extensive surgery may be needed if the lymph nodes are compromised. Melanomas with deep local invasion, or that have spread to the lymph nodes, may be treated with surgery, immunotherapy, chemotherapy, and/or radiation therapy. Advanced cases may be treated with palliative surgery, chemotherapy and/or radiation therapy, and newer targeted or immunotherapy drugs.

Melanoma is highly curable if detected in its earliest stages and treated properly (1, 2). However, melanoma is likely to spread to other parts of the body. The 5- and 10-year relative survival rates for melanoma patients are 91 and 89%, respectively. For localized melanoma (84% of cases), the 5-year survival rate is 98%; survival declines to 62 and 16% for regional and distant stage disease, respectively. Patients with tumors that have invaded the deep dermis or spread to the draining lymph nodes have a high risk of recurrence after surgery, and safe and effective therapeutic options are still limited as adjuvant treatments. The approval of oncogene-targeted drugs such as vemurafenib and dabrafenib for BRAF^V600^ mutated melanoma (3, 4), and of monoclonal antibodies (mAbs) targeting immunomodulatory molecules such as ipilimumab for CTLA-4 (5) and pembrolizumab for PD-1 (6), has dramatically changed the treatment of advanced melanoma in recent years. All of these agents have demonstrated a positive impact on overall survival (OS) and impressive clinical responses in advanced melanoma patients. However, related toxicity and emerging resistance are ongoing challenges. There is increasing evidence that a combination therapy using oncogene-targeted drugs and immunotherapy could produce long-lasting responses in a broader spectrum of patients.

In the present work, we will revisit the latest, promising oncogene-targeted drugs and several immunotherapeutic approaches that are under intense research, as well as their clinical effects on melanoma patients. We will discuss the preclinical and clinical evidence supporting the rationale for the combination of oncogene-targeted therapies and immunotherapy to improve patient outcome.

## Advances in Melanoma Treatment

### Oncogene-targeted drugs

Cutaneous melanoma has the highest mutational frequency of any tumor pathology. Tumor transformation and progression is believed to be sustained by the emergent combination of genetic changes (7). Whole-genome sequencing studies reveal that the point mutation load is proportional to UV exposure rays (C > T/G > A), with the highest rates in patients with a documented history of chronic sun exposure; however, there are also non-related sun exposure mutations (8). A recent report describing a panel of cancer mutations in clinical melanoma samples of different subtypes, including cutaneous, acral, mucosal, and unknown primary melanomas, revealed that the number of mutations per tumor was associated with melanoma subtype (9). Here, we will describe the most prevalent mutations in melanoma that resulted in development of targeted therapies, some with impressive clinical results.

The canonical MAPK signaling pathway – receptor tyrosine kinase (RTK)–RAS–RAF–MEK–ERK – promotes survival, growth, migration, and resistance to apoptosis. The MAPK pathway initiates in the cell membrane, either by ligand interaction with a RTK, or by the adhesion of integrins to the extracellular matrix. Mutations in the serine/threonine–protein kinase BRAF were identified a decade ago as the most frequent in CM, with a prevalence of ~40–60% clinical cases (10). Updated data reveal that the T1799A mutation, resulting in the BRAF^V600E^ constitutively active isoform, accounts for ~75% of BRAF^V600^ gene mutations (11). Other less frequent variants include BRAF^V600K^ and BRAF^V600R^, which are present in ~20 and ~4% of BRAF^V600^ cases. In melanoma, NRAS mutations are rare in BRAF^V600^ tumors (1.6%). Among 20% of melanoma tumors are mutated in NRAS, especially in exon 2 (NRAS^Q61R/Q61K/Q61L^). Additionally, 16% of melanoma tumors present TP53 mutations, and concurrent TP53 mutations are the most frequent events in tumors with BRAF^V600^ or NRAS mutations. BRAF^V600^ and TP53 mutations are significantly associated with cutaneous primary tumor location, while NRAS and c-KIT alterations were associated with acral and mucosal melanoma (9). Other sequencing studies indicate that 8% of MEK1 and MEK2 mutations may occur simultaneously with BRAF (60%) or NRAS (10%) mutations (12). The most frequent MEK mutants, MEK1^P124S^ and MEK1^E203K^, are related to ERK phosphorylation. All of these driver mutations lead to the constitutive activation of the MAPK pathway; thus, they arose as appropriate druggable targets with specific inhibitors.

*Vemurafenib* (PLX-4032, Zelboraf, Roche) is a small, orally bioavailable molecule that selectively binds the ATP-binding site of BRAF^V600E^ kinase and inhibits its activity (13). Vemurafenib efficacy was assessed in a randomized clinical trial against Dacarbazine in metastatic CM patients carrying the BRAF^V600E^ mutation. Vemurafenib produced a higher response rate (48 vs. 5%), and an increase in OS (84 vs. 64%) and disease-free survival (DFS) (5.3 vs. 1.6 months). Impressive rapid tumor remissions were observed, with a median time-to-response of 1.45 months (3). Skin complications were frequently associated with treatment: 24% of patients from the vemurafenib arm developed low-grade cutaneous squamous-cell carcinomas or keratoacanthomas, through paradoxical ERK activation. These tumors required excision and continuous dermatologic evaluation during treatment. Fortunately, patients with BRAF^V600^ mutations other than BRAF^V600E^ will respond to vemurafenib, including BRAF^V600K^ and BRAF^V600R^ (14, 15). Vemurafenib became one of the cornerstones of metastatic or unresectable CM treatment with its approval in 2011 by the Federal Drug Administration (FDA) and in 2012 by the European Medicines Agency (EMEA). Despite the impressive initial tumor remissions observed with vemurafenib, drug-resistance has limited the duration of remissions; therefore, great efforts are being directed toward revealing and overcoming the mechanisms of resistance to BRAF inhibition (16).

*Dabrafenib* (Tafinlar, GlaxoSmithKline) is another orally bioavailable BRAF^V600E^ small-molecule inhibitor, which was approved by the FDA and EMEA in 2013 for treatment of unresectable or metastatic CM with BRAF^V600E^ mutation. In a randomized trial of advanced CM patients with BRAF^V600E^ determined tumors, dabrafenib significantly improved progression-free survival (PFS) compared to Dacarbazine (5.1 vs. 2.7 months) (4). Although 6% of patients from the dabrafenib arm developed keratoacanthoma or squamous-cell skin carcinoma, they did not require dose modification or interruption. *Trametinib* (Mekinist, GlaxoSmithKline) is an oral MEK1 and MEK2 inhibitor targeting the MAPK pathway downstream. This inhibitor provided longer PFS than Dacarbazine and Paclitaxel (4.8 vs. 1.5 months) for CM patients with unresectable metastatic BRAF^V600E^ tumors, and was therefore approved for CM treatment by the FDA in 2013, and by the EMEA in 2014 (17). The combination of dabrafenib and trametinib proved superior to monotherapy and produced fewer side effects, which led to FDA approval in January 2014.

Upstream in the MAPK pathway, there are altered RTKs in melanoma, including c-KIT, EGFR, and PDGFR. c-KIT is involved in the development and maintenance of melanocytes, activating the MAPK, PI3K–AKT, and Janus kinases (JAK)–signal transducers and activators of transcription (STAT) proliferation and survival pathways. Amplifications or mutations in c-KIT account for 4% of melanomas, and are most frequently found in acral, mucosal, and chronically sun-damaged skin (18). Although less prevalent in Caucasian populations, these subtypes constitute approximately 65% of the melanomas observed in Asians and African American populations. A large mutational analysis of RTKs performed in metastatic CM samples revealed that growth factor receptor ERBB4 was mutated in 19% of the samples. This receptor is involved in AKT signaling, and can be down-regulated by either ERBB4 knockdown or inhibition with Lapatinib (19).

*Imatinib* (Gleevec, Novartis) is an orally available, chemical ATP-competitive RTK inhibitor, which prevents phosphorylation and the subsequent activation of growth receptors and their downstream signal transduction pathways. Its efficacy was initially demonstrated in the BCR-ABL oncogene in hematological malignancies, and in RTKs such as c-KIT and PDGFR, which are frequently altered in gastrointestinal tumors. A phase II study in metastatic melanoma patients with mutated or amplified c-KIT showed a 23% overall response rate (ORR) with imatinib therapy (20). *Dasatinib* (Sprycel, Bristol-Myers Squibb) is another synthetic broad-spectrum multi-kinase inhibitor, with potent effect on BCR-ABL, SRC, c-KIT, PDGFR, and ephrin TK. Dasatinib has demonstrated only modest clinical efficacy in melanoma patients as a single agent (21). Tumor regression was noted in approximately 14% of total patients without c-kit mutations, and in one of two patients with tumor c-kit mutations. This level of activity suggests that biomarker-based patient pre-selection, in this case by c-KIT alteration, may identify a subset of patients that can potentially derive benefit from dasatinib.

### Monoclonal antibodies

Tumor evolution is the result of continuous feedback between tumor cells and their environment. The immune system is a crucial player, since it can both repress and enable tumor growth and is capable of shifting from an anti-tumor immune environment to a tumor-permissive one, as described by the *theory of cancer immunoediting* (22). There is significant evidence of molecules and immune populations involved in tumor immunoediting in CM (23). In particular, *immune checkpoints* refer to the circuit of inhibitory pathways that the immune system uses to modulate the duration and amplitude of immune responses. Many of these signals are initiated by ligand–receptor interactions and many tumors express these ligands as a mechanism of immune escape, particularly against tumor specific T cells. However, these ligand–receptor interactions can be blocked by specific antibodies, which then halt the immune checkpoint blockade and favor an effector anti-tumor immune response. The binding of co-stimulatory B7 molecules in Antigen-Presenting Cells (APC) to the CD28 receptor on naïve T cells induces the expression of CTLA-4 (cytotoxic T-lymphocyte-associated antigen-4). The CTLA-4 molecule has a higher affinity for B7 than CD28 and has an inhibitory function, thereby helping to extinguish the signal. Another inhibitory molecule is PD-1 (Programed death-1), which is a co-receptor expressed in activated and exhausted T and B cells. Activated PD-1 negatively regulates T cell activation through the suppression of the PI3K/Akt pathway. PD-1 interacts with two ligands: B7-H1 (PD-L1), the main mediator of the immunosuppressive response, and B7-H2 (PD-L2). In tumor pathology, PD-L1 expression is increased on tumor cells and APCs, while the expression of CTLA-4 is increased on APCs and effector T cells (24, 25).

*Ipilimumab* (Yervoy, Bristol-Myers Squibb) is a monoclonal antibody (IgG1) directed against CTLA-4 that was developed for systemic anti-tumor immunotherapy. The effects of ipilimumab in unresectable melanoma patients were assessed in a randomized trial against a gp100-peptide vaccine. Patients receiving ipilimumab plus gp100-peptide vaccine showed improved OS in comparison to those receiving the vaccine alone (10.1 vs. 6.4 months), with a median time-to-response of 3.3 months. ORR after ipilimumab administration ranged from 10 to 20%. Furthermore, complete regression continued throughout the 2 years of follow-up. No differences in OS were observed between ipilimumab treatment alone and ipilimumab plus the gp100-peptide vaccine (5). Because ipilimumab stimulates T cells, there is substantial risk of immune-related adverse events (irAEs). In this trial, 10–15% of patients presented grade 3/4 irAEs, with seven related deaths. Management of irAEs included initiation of high-dose corticosteroids and discontinuation of ipilimumab. In 2011, the FDA and EMEA approved the use of ipilimumab for the treatment of unresectable or metastatic melanoma, contributing to the renaissance of immunotherapy in cancer treatment. Given the potential for toxicity, ipilimumab approval was predicated on a risk evaluation and mitigation strategy. *Tremelimumab* (CP-675206, Pfizer/MedImmune) is another recently developed CTLA-4-blocking monoclonal antibody (IgG2). Although tremelimumab generated durable responses in metastatic melanoma patients in phase I and II trials, it failed to demonstrate a survival advantage in a randomized trial against standard-of-care chemotherapy (26).

*Pembrolizumab* (formerly Lambrolizumab, Keytruda, Merck), an IgG4 monoclonal antibody against PD-1, was recently approved in September 2014 by the FDA for treatment of patients with unresectable or metastatic melanoma. Approval was based on the results obtained in a randomized trial comparing two doses of pembrolizumab in metastatic melanoma patients whose cancer had progressed following treatment with ipilimumab or targeted therapy in BRAF^V600^ tumors (NCT01295827) (6). Key exclusionary criteria included any autoimmune disease, induced immunosuppression, and/or a history of severe irAEs from treatment with ipilimumab. The ORR was 26% at both doses, with a median time-to-response of 12 months. Treatment was well tolerated; the only drug-related grade 3 adverse event was fatigue, which was reported by 3% of patients in the 2 mg/kg pembrolizumab group. Currently, there are two ongoing, randomized, confirmatory trials for pembrolizumab in advanced melanoma patients (NCT01866319, NCT02083484). *Nivolumab* (Bristol-Myers Squibb) is another antagonist monoclonal antibody (IgG4) specific to PD-1. A phase I trial in several solid tumors showed a cumulative response in 28% of melanoma patients (27). Interestingly, only patients with PD-L1-positive tumors achieved an objective response, suggesting a relationship between PD-L1 expression on tumor cells and clinical response. Only one patient experienced a serious AE, inflammatory colitis. Controlled phase III trials of nivolumab vs. standard-of-care chemotherapy with prospective survival end points are currently underway (NCT01721772, NCT01721746) in melanoma patients. In a phase I trial in metastatic melanoma patients, co-administration of nivolumab and ipilimumab, followed by nivolumab, resulted in rapid ORR in 40% of patients, with an acceptable level of AEs (28). Currently, a phase III study comparing nivolumab or nivolumab plus ipilimumab vs. ipilimumab alone in advanced melanoma patients is underway (NCT01844505). Furthermore, specific biomarkers are being studied, including baseline variations in activated and memory T cells, interferon inducible factors, and tumor T cell infiltration (CD4^+^ and CD8^+^ cells) (NCT01621490).

Another strategy for interference with the PD-1 ↔ PD-L1 axis is *BMS-936559* (Bristol-Myers Squibb), a monoclonal antibody (IgG4) targeting PD-L1. In the initial trial, BMS-936559 induced tumor regression (ORR of 6–17%) and disease stabilization (rates of 12–41% at 24 weeks) in patients with advanced cancers, including non-small-cell lung cancer, melanoma, and renal cancer (29). Serious AEs were detected in 9% patients. Other antagonist mAbs against PD-L1 are currently being assessed in clinical trials for metastatic treatment, such as *MEDI4736* (Astrazeneca, NCT01693562) and *RG7446* (Roche, NCT01375842).

### Therapeutic vaccines

Cutaneous melanoma is an immunogenic tumor; several tumor antigens (Ags) have been identified and evidence of tumor immunoediting has been observed in patients (23). Immunotherapy has emerged as an option of interest for CM treatment. Immunotherapy seeks to stimulate, restore, manage, and even complement the patient’s own immune system to control tumor growth and dissemination. Among other immunotherapeutic approaches, vaccines could be administered as an adjuvant therapy after tumor excision, with the purpose of eliciting long-lasting immunity and controlling micro-metastatic foci. The rational basis for such vaccines is that tumor Ags must be captured by APCs, which migrate to the lymph nodes where they further activate CD4^+^ and CD8^+^ cells. This triggering of the adaptive immune response would also result in the development of immunological memory. The main objectives of active immunotherapy against melanoma are to overcome the immunosuppression produced by the tumor and its microenvironment, to stimulate specific immune effectors that can destroy tumor cells, and to increase immunogenicity to tumor Ag. Pre-existing anti-tumor T cells may be ineffective at rejecting the tumor either because their frequency is too low, because tumor cells were selected to escape recognition, or because such lymphocytes are functionally deficient. Vaccination can induce cytokine cascades both locally and systemically, resulting in the activation and proliferation of anti-melanoma Ag precursors, and infiltration of effector immune populations into tumors. In general, vaccines require two critical components, the Ag source and the adjuvant (30). Therapeutic vaccines include the use of different Ag sources, such as peptides, proteins, nucleic acids, tumor lysates, recombinant virus, or whole irradiated cells. Alternatively, dendritic cell (DC) vaccines are comprised of patients’ DC *in vitro* loaded with an Ag source, *ex vivo* matured and then given back to the patient.

Peptide vaccines are directed at one or several representative CM Ag. To induce T cell responses, it is necessary to immunize with adjuvants or Toll-like receptor (TLR) ligands. Numerous clinical trials were conducted with this strategy, and although some encouraging results were observed, peptide vaccines promote the selection of tumor Ag-negative resistant populations. Peptide Ags emulsified in incomplete Freund’s adjuvant (IFA) are widely used to vaccinate cancer patients. While several clinical trials testing peptide/IFA-based vaccines have documented an increase in circulating Ag-specific T cells, objective therapeutic benefits are rare (31, 32). This may be due to the presence of large peptide deposits that are protected from degradation and can prime robust specific CD8^+^ T cell responses, which are detectable in circulation but not within tumor lesions. Thus, Ag persistence at the vaccination site can create a T cell graveyard in which specific T cells accumulate and compete with the tumor site (33). It is clear that other adjuvants need to be evaluated to improve peptide delivery and immune cell stimulation.

Therapeutic vaccines comprised of whole cells or cell lysates allow the immune system to interact with a broad antigenic repertoire. This is an important consideration, since tumors are heterogeneous and CM is not an exception (34). A meta-analysis combining results from 173 immunotherapy clinical trials of several cancers found that patients immunized with whole-tumor Ag, including irradiated tumor cells, modified tumor cells, tumor extracts, tumor mRNAs, and DC pulsed with tumor extracts, showed an 8.1% clinical response compared with 3.6% in patients immunized with synthetic or recombinant peptides and proteins (35). Whole-cell vaccines are still in clinical trials. The GVAX vaccine (36), consisting of irradiated genetically modified cell lines that secrete granulocyte–macrophage colony stimulating factor (GM-CSF, Molgramostim), is currently being assayed in stage IIB-IV melanoma patients in a phase I study (NCT01435499). We have tested a vaccine comprised of allogeneic irradiated CM cells (VACCIMEL) plus BCG as an adjuvant, combined with i.d. injection of GM-CSF to promote the local attraction of monocytes and their subsequent differentiation into DC. In the Phase I study, VACCIMEL shown to be safe, and with a maximum follow-up of 139 months, mean DFS and OS have not yet been reached for stage II/III vaccinated patients (DFS and OS 58.3%) (José Mordoh, personal communication 2014); all stage IV patients progressed (37). Currently, the CSF-470 vaccine, which adds a fourth irradiated melanoma cell line to the VACCIMEL formulation, is being tested with BCG and GM-CSF against medium dose IFN-alpha 2b in a Phase II–III clinical trial in stage IIB, IIC, and III CM patients (NCT01729663).

Dendritic cell-vaccines consist of autologous treatments, in which DC precursors are purified from patients and are loaded *in vitro* with tumor Ag (irradiated cells), matured *ex vivo*, and then re-infused into the patient (38). In 2010, the FDA approved Sipuleucel-T, the first DC-based vaccine for the treatment of metastatic castrate resistant, hormone refractory prostate cancer (39). Sipuleucel-T (Provenge) is made of an autologous DC-vaccine preparation loaded with Prostatic acid phosphatase Ag and GM-CSF. In the case of CM, a phase I–II trial was conducted for stage IV patients in which autologous DC were pulsed with cocktail of synthetic five melanoma-associated peptides (gp100, tyrosinase, MAGE-A2, MAGE-A3, and MART-1 or MAGE-A1) restricted to HLA-A2 or HLA-A24, and KLH as adjuvant (40). Clinical responses were observed and OS increased: 13.6 vs. 7.3 months (vaccinated vs. non-vaccinated), and 21.9 vs. 8.1 months (high vs. low ELISPOT). Additionally, the presence of the MAGE-A1 auto-antibody before vaccination was determined as a positive prognostic factor. Other clinical studies have used autologous DC loaded with peptides, tumor cell lysates (41), killed melanoma cells (42), or tumor Ag mRNA electroporation (43), but overall clinical responses remained disappointingly low. However, the induction of specific immune responses to tumor Ags has been described. Several years ago, we demonstrated that autologous DC can capture apoptotic-necrotic, gamma-irradiated, allogeneic melanoma cells, inducing the maturation and subsequent migration of DC in response to the lymph node homing CCL19 chemokine, both in a murine model (44) and with DC isolated from peripheral blood monocytes from healthy donors (45). We conducted a Phase I clinical trial for 16 CM patients. In that study, we observed gp100 and MART-1 specific CD8^+^T lymphocytes through ELISPOT and tetramer analysis in five HLA-A*0201 patients before and after vaccination (46). In an updated analysis with a maximum follow-up of 132 months, 88.9% stage II–III patients still show no evidence of melanoma recurrence (José Mordoh, personal communication 2014).

Dendritic cell-based vaccine design has been variable, it is difficult to assess the best inoculation site, the optimal number of DCs to elicit effective immune responses, the Ag-loading strategies and how to monitor immune effector recruitment in the tumor microenvironment. A recent review indicated that DC-based cancer vaccines can elicit adaptive and innate anti-tumor immunity in at least half of all patients; however, clinical responses remain disappointingly scarce (47). The observed dissociation between clinical objective response and the prolongation of OS in some patients indicates that alternative surrogate endpoints should be used to assess the therapeutic effectiveness of DC-based immunotherapy. DC-based vaccines can positively affect the clinical outcome in the form of increased patient survival rather than the induction of tumor objective responses. The clinical benefit of DC-based immunotherapy is real but small. With 8.5% of melanoma patients achieving an objective response, DC therapy has comparable efficacy to Dacarbazine, the standard-of-care chemotherapy, and to ipilimumab, to which 5–15% of patients have an objective response (47). Today, there are no surrogate markers indicative of relevant anti-tumor immunization to evaluate vaccine efficacy, meaning that DFS and OS are the parameters that define vaccine success. Furthermore, there are no predictive markers indicating anti-tumor immune competence that could identify the patients most likely to benefit (47).

### Adoptive cell therapy

Adoptive Cell Therapy involves the administration of autologous *ex vivo*-expanded tumor-reactive T lymphocytes to properly preconditioned recipients. T lymphocytes can be expanded from tumor infiltrating lymphocytes (TIL) *ex vivo* from a patient’s own metastasis after IL-2 and anti-CD3 antibody stimulation. The expanded T cells are then re-administered after chemotherapy-induced lymphodepletion of the patient. Such melanoma TILs recognize their autologous tumor in >75% of patients. Although adoptive cell therapy (ACT) is typically administered only once, clinical responses can endure for years. The largest published description of ACT with significant follow-up showed an ORR of 56% in 93 patients who had followed a variety of lymphodepleting regimens, 19 patients (21%) had ongoing complete responses (CR) at 5–9 years of follow-up (48). Other investigators have published their experiences with ACT and reported a preliminary ORR of approximately 50%, including some CR (49–51).

### Other immunotherapies

The first immunotherapeutic drug approved for treatment of metastatic melanoma was recombinant Interleukin-2 or aldesleukin (Proleukin, Prometheus Laboratories), which promotes activation of T, B, and NK-cells. Studies indicate that high doses of IL-2 (HD IL-2) induced long-term responses in 16% of patients and a CR in 6% of cases (52). IL-2 provided the first “proof of principle” that a drug targeting the immune system can control melanoma at long-term. However, HD IL-2 is very toxic. Serious AEs can occur, such as capillary leak syndrome, breathing problems, serious infections, seizures, allergic reactions, and heart problems among other possible complications (53).

Later on, Interferon alpha 2b (IFN-α 2b) was approved by the FDA as a post-surgical adjuvant treatment. Previous studies have shown that adjuvant treatment with IFN-α2b at intermediate doses for 2 years increased DFS of melanoma patients with high risk of recurrence (stages IIb, IIC and III) in 10%, with no significant effect on OS (54). IFN-α 2b is associated with severe side effects. Based on the results of the EORTC 18991 trial, the FDA approved PEG-interferon α-2b (PEG-IFN) (Sylatron) as adjuvant therapy for high-risk melanoma. The median PFS of the PEG-IFN group was significantly longer than the observation group, while OS, a secondary endpoint, was not significantly different between the two groups. One-third of the patients receiving PEG-IFN discontinued treatment because of toxicity. PEG-IFN is characterized by a longer half-life and can be administered subcutaneously. Much progress has been made in unraveling the mechanisms of action of IFN’s anti-tumor activity. These include cell cycle inhibition by G1 arrest and anti-proliferative activity (55), the induction of apoptosis (56–58), and the reduction of angiogenesis in some tumors by reducing basic fibroblast growth factor (bFGF) and vascular endothelial growth factor (VEGF) production (59, 60). Several indirect immunomodulatory effects of interferon treatment have been identified, including a role in T cell differentiation and B cell development, which may aid in immune surveillance by increasing Ag processing and upregulating MHC-I expression, thus facilitating Ag recognition by cytotoxic CD8^+^ T cells. Besides, IFN-α produces strong activation of monocytes and macrophages. Activated macrophages produce reactive oxygen species and reactive nitrogen intermediates, which have cytotoxic effects on target cells (61). Additionally, activated monocytes produce cytokines that initiate a Th1 response. More recently, IFN-α has been shown to induce activation of NK-cells through the increase of NKG2D and CD161 stimulatory signaling, thus enhancing NK-cell killing of tumor cells (62, 63).

### Lights and shadows of therapeutic approaches for melanoma treatment

The phenomenon of oncogene-addicted tumors allowed the development of targeted therapies with impressive clinical results and, for the first time, rapid remissions in advanced metastatic melanoma patients. These results supported the approval of targeted therapies. However, targeted therapies are only useful for patients whose tumors possess specific mutations. There can be serious related AEs, and the emergence of escape mechanisms and drug-resistance may be difficult to overcome.

The unraveling of key immunomodulatory pathways involved in anti-tumor immune responses has allowed the development and approval of specific therapeutic approaches. For instance, immune checkpoint-specific antagonist mAbs allow the achievement of long-lasting clinical responses in a small proportion of advanced metastatic patients; these responses are often slow to manifest and take several months to achieve (5). Furthermore, there are concerning irAEs, primarily because these therapies are directed toward targets that are also involved in physiological processes. Additionally, specific biomarkers are needed to implement these treatments in the patient populations that would benefit the most.

Melanoma therapeutic vaccines have shown to be safe and to preserve the quality of life of patients with cancer (64, 65). Specific anti-tumor Ag responses have been demonstrated in about half of vaccinated patients. A small number of patients documented clinical OR (~8.5%) (66, 67). The most rational application of therapeutic vaccines seems to be as an adjuvant therapy aimed at the control of micro-metastatic dissemination. However, there is insufficient evidence supporting melanoma vaccine efficacy, or any correlation between immune responses and clinical objective responses. Most clinical studies have tested these vaccines in metastatic patients, perhaps foreshadowing possible control in earlier stage melanoma recurrence. Immune monitoring in the tumor microenvironment is difficult to assess and relevant immune effectors have not yet been identified.

Long-term responses to cytokine therapies such as IL-2 and IFN-α have been documented, though in very few patients, highlighting once again the intimate relationship between the immune system and melanoma (52, 54). However, the impact of cytokine therapy on patients’ OS has not been demonstrated and the associated AEs remain a major problem. Additional research is needed to discover appropriate markers that allow the identification of the patients that are most likely to respond to these therapies.

The ORR of ACT is approximately 50% (51). Some CR have been reported, suggesting that this strategy may be an attractive alternative for some melanoma patients. However, this procedure is expensive, only 30–40% of biopsy specimens yield satisfactory T cell population recovery for TIL preparation, and the process is laborious and time intensive (49). Furthermore, prior host conditioning with chemotherapy is required to increase the response to ACT. This conditioning is associated with serious AEs, including opportunistic infections and the frequent induction of vitiligo and uveitis, presumably due to autoimmunity (49).

The absence of an association between objective responses and OS has been widely reported with immunotherapeutic drugs and targeted therapies. Immunotherapies often produce an atypical clinical response pattern that includes delayed initial increases in tumor burden, which is associated with inflammation or immune cell infiltration of the tumor lesions, followed by regression and changes in disease progression kinetics (68). Altogether, these data underscore the idea that cancer immunotherapies need alternative efficacy endpoints in addition to the traditional outcome parameters used in oncology clinical trials.

## Immunotherapy and Oncogene-Targeted Drugs: The Power of Combination Strategies

As we have seen, new melanoma treatments, including oncogene-targeted therapies and immunotherapies, have been recently approved. Due to their different modes of action and possible interactions, a special interest has arisen in using combination therapies to improve melanoma control. Targeted therapies can achieve impressive and rapid tumor remissions, though these results are shadowed by the emergence of resistance mechanisms through selection in heterogeneous tumors, limiting clinical response to a relatively short duration. In contrast, immunotherapy can give rise to long-term melanoma control by eliciting active immune effectors that may lead to curative responses, but in a small, select group of patients.

Why combine oncogene-targeted therapies and immunotherapy? There is increasing evidence that oncogenes play a role in the modulation of the expression of immune regulatory genes and therefore interfere with the immune microenvironment. We will focus on BRAF^V600^ in melanoma, where there is the most evidence. Initial reports showed that BRAF^V600E^ CM cell lines secreted immunosuppressive IL-10, VEGF, and IL-6 molecules and suppressed IL-12 and TNF-α production by DC *in vitro*, and that this could be reversed by MEK-inhibitors or specific siRNA for BRAF^V600E^ or STAT-3 (69). Human BRAF^V600E^ CM cell lines also induced the expression of IL-1α and IL-1β, which in turn induced COX-2 and PD ligand expression in tumor-associated fibroblasts, suppressing T cell function. Interestingly, IL-1α expression was down-regulated in human tumor biopsies following vemurafenib treatment (70).

Studies investigating the underlying mechanisms of action describe an increase in CD4^+^, CD8^+^/Treg, and NK-cell numbers following PLX-4720 activity in BRAF^V600E^-driven murine melanoma models, showing that host immunity greatly contributes to inhibitor activity. Furthermore, the combination of PLX-4720 and anti-CCL2 or agonistic anti-CD137 antibodies improved anti-tumor responses (71). Regarding innate immunity, NK-cells will play a critical role in the control of BRAF^V600E^ metastatic melanoma tumors treated with PLX-4720 through a perforin-dependent pathway. In the context of IL-2, PLX-4720-induced ERK1/2 phosphorylation, CD69 expression and IFN-γ release post NKp30 ligation in human NK-cells. A corollary from this work was that simultaneous inhibition by BRAF and MEK might preclude the activation of BRAF^WT^ NK-cells. A low dose of IL-2 improved the anti-metastatic efficacy of PLX-4720, supporting the combination of NK-cell stimulatory agents with BRAF-inhibitors (72). With regards to adaptive immunity, BRAF^V600E^ inhibition reportedly increased the expression of melanocyte differentiation Ags on CM cells and enhanced the activation of Ag-specific T cells *in vivo* (73). Neither PLX-4720 nor PLX-4032 BRAF^V600E^ specific inhibitors interfered with the viability or the functionality of T cells, allowing the implementation of a combinatorial approach with immunotherapy (74). Other works reported that BRAF-inhibitors induced a paradoxical MAPK-dependent functional activation of T cells (75). This effect was dose-dependent on BMS-908662, a pan-RAF inhibitor (with activity against B-RAF^V600^, B-RAF^wt^, A-RAF, and C-RAF), and dose-independent on PLX-4720 inhibitor. The authors proposed that the paradoxical ERK activation in BRAF^wt^ cells may be a common event with ATP-competitive inhibitors of the BRAF kinase domain and showed that MEK inhibition blocks this paradoxical activation. Interestingly, the combination of BMS-908662 with a CTLA-4 blockade improved anti-tumor action. In patients, reports indicated that BRAF-inhibitors induced tumor Ag expression and the infiltration of CD8^+^ and CD4^+^ T cells in metastases shortly after the initiation of treatment, which was followed by a reduction in tumor size. Progressing patients, however, showed a decrease in TILs. These results further support combinatorial strategies (76). Other analysis of tumor biopsies pre- and post-treatment with combinatorial dabrafenib/trametinib treatment showed a reduction of the immunosuppressive cytokines IL-6 and IL-8. However, CD8^+^ tumor infiltration, along with PD-1, Tim-3, and PD-L1 expression also increased following treatment, suggesting that these oncogene-targeted therapies might be limited and should be combined with immunotherapy to achieve long-term responses (77).

There remains much to learn about how the exposure of different classes of immune cells to vemurafenib modulates immune system activity. Interestingly, two ongoing clinical studies are currently analyzing the kinetics and effects of BRAF inhibition with vemurafenib (960 mg BID) on the innate and adaptive immune system in patients with unresectable melanoma expressing a BRAF^V600^ mutation (NCT01942993 and NCT01813214). These studies will evaluate changes in the immune cellular signature in blood circulation, comparing the baseline to different time points after the initiation of vemurafenib treatment through immunofluorescence and flow cytometry on blood samples. Additionally, the timeline of the vemurafenib-induced increase in T cell infiltration of tumors will be established through analysis of biopsies. As will other immune-related parameters, such as the activation state of TILs, expression of immune-inhibitory proteins (B7-H1/PD-L1, IDO, Arginase), changes in endothelial homing receptor ligands and tumor-associated chemokines, the presence of immune/inflammatory expression patterns, the presence of tissue-specific destruction and IFN-gamma upregulation, and *in vitro* determination of tumor cell lysis in comparison to allogeneic tumor cells. These studies will provide a systematic and valuable body of data that will aid in understanding the immune modulation induced by anti-BRAF therapy. And will help to develop more rationalized combination strategies with BRAF-targeted therapies and immunotherapy.

## Currents Avenues of Research

In Table 1, we present a selected list of ongoing clinical studies that are exploring different combination therapies involving oncogene-targeted therapy with different immunotherapeutic approaches (current as of November 2014, source Clinical trials.gov and EMEA data base).

**Table 1:** **Ongoing clinical studies combining immunotherapy and oncogene-targeted therapy**.

Trial identifier (status)	Combination therapy	Patient condition	Study phase	Sponsor	Study title	Study design
**1. IMMUNE CHECKPOINT BLOCKADE**						
NCT01940809 (recruiting)	Dabrafenib; ipilimumab; trametinib	Stage IV melanoma or unresectable; Stage III melanoma	I	National Cancer Institute (NCI)	A Sequential Safety and Biomarker Study of BRAF-MEK Inhibition on the Immune Response in the Context of CTLA-4 Blockade for BRAF Mutant Melanoma	**Arm A (ipilimumab, dabrafenib, trametinib):** Patients receive dabrafenib PO BID and trametinib PO QD for 25 days. Patients then receive ipilimumab IV over 90 min. Treatment with ipilimumab repeats every 3 weeks for four courses in the absence of disease progression or unacceptable toxicity. **Arm B (ipilimumab, trametinib):** Patients receive trametinib PO QD for 25 days. Patients then receive ipilimumab IV over 90 min. Treatment with ipilimumab repeats every 3 weeks for four courses in the absence of disease progression or unacceptable toxicity. **Arm C (ipilimumab, dabrafenib):** Patients receive dabrafenib PO BID for 25 days. Patients then receive ipilimumab IV over 90 min. Treatment with ipilimumab repeats every 3 weeks for four courses in the absence of disease progression or unacceptable toxicity. **Arm D (ipilimumab):** Patients receive ipilimumab IV over 90 min. Treatment repeats every 3 weeks for four courses in the absence of disease progression or unacceptable toxicity. Other: laboratory biomarker analysis.
NCT01767454 (recruiting)	Dabrafenib; trametinib; ipilimumab	Solid tumors	I	GlaxoSmithKline	Phase 1 Study of the BRAF-Inhibitor Dabrafenib ± MEK Inhibitor Trametinib in Combination With Ipilimumab for V600E/K Mutation-Positive Metastatic or Unresectable Melanoma	**Doublet arm: Cohort A1** dabrafenib (150 mg orally BID) for 2 weeks + ipilimumab. **Cohort A-2** dabrafenib (100 mg orally BID) for 2 weeks + ipilimumab (3 mg/kg Q3W, four infusions) over 12–16 weeks. Dabrafenib will be continued through combination with ipilimumab and post-ipilimumab phases, until no longer of clinical benefit, unacceptable toxicity or death. **Triplet arm** Initiated using dabrafenib and ipilimumab doses established in the doublet dose-finding study. Dabrafenib and trametinib are taken orally for 2 weeks followed by ipilimumab 3 mg/kg Q3W (four total infusions over 12–16 weeks). **Cohort B-1:** dabrafenib 100 mg BID + trametinib 1 mg once daily + ipilimumab. **Cohort B2:** dabrafenib 150 mg BID + trametinib 1 mg once daily + ipilimumab. **Cohort B3:** dabrafenib 150 mg BID + trametinib 2 mg once daily + ipilimumab. Dabrafenib and trametinib will be continued through combination with ipilimumab and post-ipilimumab phases until no longer of clinical benefit, unacceptable toxicity or death.
NCT01673854 (not recruiting)	Ipilimumab; vemurafenib	Previously untreated, metastatic melanoma with activating BRAFV600 mutation	II	Bristol-Myers Squibb	A Single Arm Open-Label Phase II Study of Vemurafenib Followed by Ipilimumab in Subjects With Previously Untreated V600 BRAF Mutated Advanced Melanoma	Vemurafenib followed by Ipilimumab. **Vem 1 Phase:** vemurafenib 960 mg orally twice daily for 6 weeks followed by ipilimumab 10 mg/kg intravenous injection once every 3 weeks for four doses, then once every 12 weeks starting at week 24 until disease progression (PD) or unacceptable toxicity (for a maximum treatment period of 3 years from the first dose). **Vem 2 Phase:** vemurafenib re-started at time of PD, unacceptable toxicity on ipilimumab until PD or unacceptable toxicity.
NCT02200562 (not yet recruiting)	Ipilimumab; dabrafenib	Stage III or IV, BRAF^V600E/K/R^ positive Melanoma	I/II	University of Utah	Ipilimumab and Dabrafenib in the 1st Line Tx of Unresectable Stage III/IV Melanoma	**Experimental:** concurrent ipilimumab and dabrafenib as first line treatment in Stage III or IV melanoma.
NCT01738139 (recruiting)	Imatinib mesylate; ipilimumab	Advanced cancers: KIT confirmed GIST, melanoma, and uncategorized solid tumors	I	M.D. Anderson Cancer Center	A Phase I Trial of Ipilimumab (Immunotherapy) and Imatinib Mesylate (c-Kit Inhibitor) in Patients With Advanced Malignancies	**Ipilimumab + Imatinib Mesylate** **Dose escalation:** initial daily oral administration of imatinib mesylate (400 mg) for 14 days. A single ipilimumab treatment (1 mg/kg) given on day 15 will be added to daily to imatinib mesylate therapy. The dose escalation group’s first study cycle is 35 days. Each cycle after that is 21 days. **Expansion cohort:** using the MTD determined by the dose escalation study to treat patients with KIT confirmed GIST, melanoma, and uncategorized solid tumors. Both studies will consist of a screening visit and continuous 21-day treatment cycles. Cycles will be repeated every 21 days for four cycles until disease progression or development of intolerable toxicities, followed by a post-treatment visit.
NCT02224781 (not yet recruiting)	Nivolumab; ipilimumab; dabrafenib; trametinib	Recurrent *BRAF^V600^ Mutant* Melanoma; Stages IIIA, IIIB, IIIC and IV	III	National Cancer Institute (NCI)	A Randomized Phase III Trial of Dabrafenib + Trametinib Followed by Ipilimumab + Nivolumab at Progression vs. Ipilimumab + Nivolumab Followed by Dabrafenib + Trametinib at Progression in Patients With Advanced BRAF^V600^ Mutant Melanoma	**Arm A (immunotherapy):** IMMUNOTHERAPY INDUCTION (COURSES 1–2): Patients receive nivolumab IV over 60 min and ipilimumab IV over 90 min on days 1 and 22. Treatment repeats every 6 weeks for two courses in the absence of disease progression or unacceptable toxicity. IMMUNOTHERAPY MAINTENANCE (COURSES 3–14): Patients receive nivolumab IV over 60 min on days 1, 15, and 29. Treatment repeats every 6 weeks for up to 12 courses in the absence of disease progression or unacceptable toxicity. Upon disease progression, patients cross over to Arm C. **Arm B (BRAF-inhibitor therapy):** Patients receive dabrafenib PO BID and trametinib PO daily on days 1–42. Courses repeat every 6 weeks in the absence of disease progression or unacceptable toxicity. Upon disease progression, patients cross over to Arm D. **Arm C (BRAF-inhibitor therapy):** Patients receive dabrafenib PO BID and trametinib PO daily on days 1–42. Courses repeat every 6 weeks in the absence of disease progression or unacceptable toxicity. **Arm D (immunotherapy):** IMMUNOTHERAPY INDUCTION (COURSES 1–2): Patients receive nivolumab IV over 60 min and ipilimumab IV over 90 min on days 1 and 22. Treatment repeats every 6 weeks for two courses in the absence of disease progression or unacceptable toxicity. IMMUNOTHERAPY MAINTENANCE (COURSES 3–14): Patients receive nivolumab IV over 60 min on days 1, 15, and 29. Treatment repeats every 6 weeks for up to 12 courses in the absence of disease progression or unacceptable toxicity. Other: laboratory biomarker analysis, quality-of-life assessment.
NCT02027961 (recruiting)	Anti-PD-L1 Mab (MEDI4736); dabrafenib; trametinib	Unresectable Stage IIIc or Stage IV melanoma	I	MedImmune LLC	A Phase 1 Open-label Study of Safety and Tolerability of MEDI4736 in Subjects With Metastatic or Unresectable Melanoma in Combination With Dabrafenib and Trametinib or With Trametinib Alone	**Cohort A** (BRAF V600E or V600K mutation-positive): Dabrafenib/Trametinib/MEDI4736. **Cohort B** (BRAF mutation-negative) Trametinib/MEDI4736. **Cohort C** (BRAF mutation-negative): Trametinib/MEDI4736. Study evaluation will include maximum tolerated dose, safety, anti-tumor activity, pharmacokinetic, and immunogenicity of MEDI4736.
NCT02130466 (recruiting)	Pembrolizumab; dabrafenib; trametinib	Advanced melanoma (unresectable Stage III) or metastatic (Stage IV) excluding uveal, mucosal, or ocular melanoma	I/II	Merck Sharp & Dohme Corp. in collaboration with Glaxo Wellcome	A Phase I/II Study to Assess the Safety and Efficacy of MK-3475 in combination with Trametinib and Dabrafenib in subjects with advanced melanoma	**Pembrolizumab + Dabrafenib + Trametinib** (Parts 1, 2, and 3) Pembrolizumab intravenously (IV) on Days 1 and 22, or on Days 1, 15, and 29 of each 6-week cycle; dabrafenib capsules, 150 mg/day total, orally, in a divided dose (twice per day, or BID) starting on Day 1, through study treatment discontinuation; trametinib tablets, 2 mg, orally, once daily (QD) starting on Day 1, through study treatment discontinuation. **Placebo Comparator: Placebo + Dabrafenib + Trametinib** (Part 3) Placebo IV on Days 1 and 22, or on Days 1, 15, and 29 of each 6-week cycle; dabrafenib capsules, 150 mg/day total, orally, in a divided dose BID starting on Day 1, through study treatment discontinuation; and trametinib tablets, 2 mg, orally, QD starting on Day 1, through study treatment discontinuation. **Pembrolizumab + Trametinib** (Parts 1 and 2) Pembrolizumab IV on Days 1 and 22, or on Days 1, 15, and 29 of each 6-week cycle and trametinib tablets, 2 mg, orally, QD starting on Day 1, through study treatment discontinuation. **Pembrolizumab + Dabrafenib** (Parts 1 and 2) Pembrolizumab IV on Days 1 and 22, or on Days 1, 15, and 29 of each 6-week cycle and dabrafenib capsules, 150 mg/day total, orally, in a divided dose BID starting on Day 1, through study treatment discontinuation.
NCT01245556 (completed)	BMS-908662 ipilimumab	Unresectable stage III or metastatic melanoma with V600E mutation	I	Bristol-Myers Squibb	A Phase 1 Study of a RAF inhibitor (BMS-908662) administered in combination with immunotherapy (ipilimumab) in subjects with unresectable Stage III or Stage IV melanoma	**Experimental: BMS-908662 or Ipilimumab (A):** BMS-908662, oral, escalating doses starting at 25 mg, Q12h daily, continuously. Ipilimumab, IV, escalating doses starting at 3 mg/kg. Once every 3 weeks for 6 weeks, then once every 12 weeks, continuously. **Experimental: BMS-908662 or Ipilimumab (B):** BMS-908662, oral, escalating doses starting at 25 mg Q12h daily for 3 weeks with 3 weeks interval for four cycles, then Q12h daily for 3 weeks every 12 weeks, continuously. Ipilimumab, IV, escalating doses starting at 3 mg/kg, Once every 6 weeks for four cycles, then once every 12 weeks, continuously.
**2. VACCINES**						
NCT01876212 (recruiting)	Dasatinib; DC vaccine	Metastatic melanoma	I	Hussein Tawbi, University of Pittsburgh	A randomized Phase 2 Pilot Study of Type I-polarized autologous dendritic cell vaccines incorporating tumor blood vessel antigen (TBVA)-derived peptides in combination with dasatinib in patients with metastatic melanoma	**Arm A:** vaccine (10^7^ cells, i.d.) prior dasatinib (orally, 70 mg 2 × day). **Arm B:** vaccine (10^7^ cells, i.d.) concomitant dasatinib (orally, 70 mg 2 × day). Vaccine i.d administration will be in the vicinity of the four nodal drainage groups of the four extremities.
NCT02077114 (completed 9/25/2014)	Vaccine-peptide derived from the protein IDO (IDO Long); ipilimumab; vemurafenib	Malignant melanoma with metastasis	I	Herlev Hospital	Peptide vaccination in combination with either ipilimumab or vemurafenib for the treatment of patients with unresectable Stage III or IV malignant melanoma A Phase I study (first in man)	**Experimental: Ipilimumab** Patients who are candidates for treatment with Ipilimumab according to standard criteria. **Experimental: Vemurafenib** Patients are candidates for treatment with Vemurafenib who according to standard criteria. Vaccine consisting of a peptide derived from the protein IDO. All patients will receive seven vaccines containing IDO long.
**3. OTHER IMMUNOTHERAPIES**						
NCT01754376 (recruiting)	Vemurafenib; aldesleukin	Metastatic or unresectable melanoma with V600E mutation	II	Massachusetts General Hospital	COMBAT 1: A Phase II trial of combined BRAF-targeted therapy and immunotherapy for melanoma	**Treatment arm:** Oral vemurafenib twice a day plus i.v infusion of aldesleukin. Vemurafenib twice a day for 2 weeks followed by one course of aldesleukin via IV infusion on Day 15 (aldesleukin via IV infusion every 8 h for the first 5 days (week 1); one course of aldesleukin is 12 weeks long. During days 29–33 one more week of aldesleukin (Week 2). Oral vemurafenib twice daily will continue during the course of aldesleukin.
NCT01683188 (recruiting)	Vemurafenib; HD IL-2 (Proleukin)	Metastatic melanoma	IV	Prometheus Laboratories	A Multi-Center Study of High-Dose Aldesleukin (Interleukin-2) + Vemurafenib therapy in patients with BRAFV600 mutation-positive metastatic melanoma	**Cohort 1:** Patients who have received fewer than 7 weeks vemurafenib dosing prior to treatment with HD IL-2. Drug: vemurafenib + HD IL-2 **Cohort 2:** Patients who have receive >7–18 weeks vemurafenib dosing prior to treatment with HD IL-2.
NCT01959633 (recruiting)	Vemurafenib; Peg-interferon	Unreseactable stage IIIb-IV metastatic melanoma, V600 BRAF mutations positive	I/II	Fondazione Melanoma Onlus	Phase I-II study of the combination vemurafenib plus PEG-interferon in advanced melanoma patients harboring the V600BRAF mutation	Vemurafenib 960 mg BID + Peg-interferon 1/2/3 μg/kg once weekly. **Phase I:** Cohort (1) Peg-interferon 1 μg/kg once per week s.c. Cohort (2) Peg-interferon 2 μg/kg once per week s.c. Cohort (3) Peg-interferon 3 μg/kg once per week s.c. Interferon treatment should start after 15 days of vemurafenib only. **Phase II:** Is included the cohort selected by phase I due to MTD and expanded at RD. Objectives: verify if the combination vemurafenib plus PEG-interferon in advanced melanoma patients harboring the BRAF^V600^ mutation is more active than vemurafenib and to verify the upregulation of IFNAR1 expression in patients treated with the combination vemurafenib plus PEG-interferon.
NCT01585415 (active not yet recruiting)	Vemurafenib; young TIL; cyclophosphamide; fludarabine; aldesleukin	Metastatic Cancer; Melanoma	I	National Cancer Institute (NCI)	A Pilot Trial of the Combination of Vemurafenib With Adoptive Cell Therapy in Patients With Metastatic Melanoma	Patients will undergo biopsy or resection to obtain tumor for generation of autologous TIL cultures. When cryopreserved TIL are available patients will begin the administration of Vemurafenib 960 mg (day 1) twice daily until the disease progresses or patients are taken off protocol. On day 7, patients will begin a non-myeloablative lymphocyte depleting preparative regimen of cyclophosphamide (60 mg/kg/day IV) on days 7 and 6 and Fludarabine (25 mg/m^2^/day IV) on days 5 through 1. On day 0, patients will receive between 1 × 10^9^ and 2 × 10^11^ young TIL and then begin high-dose aldesleukin (720,000 IU/kg IV every 8 hours for up to 15 doses). Clinical and immunologic responses will be evaluated about 4-6 weeks after the last dose of aldesleukin.
NCT01659151 (recruiting)	Vemurafenib; HD IL-2; ACT with TIL infusion; lymphodepletion (fluradabine and cyclophosphamide)	Metastatic melanoma	II	H. Lee Moffitt Cancer Center and Research Institute	A Phase II clinical trial of vemurafenib with lymphodepletion plus adoptive cell transfer and high-dose IL-2 in patients with metastatic melanoma	Lymphodepletion regimen with fludarabine and cyclophosphamide will be given before TILs infusion. ACT with TIL infusion: TILs obtained from surgically removed tumors will be amplified *ex vivo* and then given back to the patient by i.v. infusion. A high-dose regimen of IL-2 will be given after participants receive the infusion of the T cells. Vemurafenib will be given for about 3 weeks while T cells are being grown in the lab and then again after T cell infusion for up to 2 years.
NCT01758575 (recruiting)	Sunitinib; sorafenib; pazopanib; cetuximab; panitumunab; everolimus; vemurafenib; ipilimumab	Advanced or metastatic solid tumors	Observational	VU University Medical Center	Clinical evaluation of the underlying mechanisms of targeted therapy-related toxicities	**Antiangiogenic tyrosine kinase inhibitors** Sunitinib: 50 mg orally, daily. Sorafenib: 400 mg orally, twice daily Pazopanib: 800 mg orally, daily. **EGFR inhibitors** Cetuximab 250 mg/m^2^ intravenously, weekly. Panitumumab 6 mg/kg intravenously, every 2 weeks. **mTOR inhibitors** Everolimus 10 mg orally, daily. BRAF-inhibitor Vemurafenib 960 mg orally, twice daily. **Anti-CTLA-4 antibody** Ipilimumab 3 mg/kg intravenously, every 3 weeks.

### Immune checkpoint blockade and oncogene-targeted therapies

Expectations are high regarding immune checkpoint blockade and oncogene-targeted combination therapy, largely because of their individual success in the treatment of advanced melanoma *per se*, but also due to previous studies that have described oncogene modulation of the immune microenvironment. Several initial phase I/II trials are being conducted to evaluate the safety, quality of life, and immune functional status changes, in order to define an administration schedule and related biomarkers for further clinical development. These trials usually exclude patients with active autoimmune disease or known immune impairment; for example, patients receiving systemic immunosuppression for organ transplantation.

Surprisingly, a study with ipilimumab and vemurafenib in metastatic BRAF^V600^ mutated CM patients had to be terminated due to hepatic toxicities (NCT01400451) (78). Patients received a running period of oral vemurafenib, and then concomitant vemurafenib with intravenous ipilimumab. In both cohorts, most patients developed grade 2/3 hepatic AEs, including elevation in aminotransferase and total bilirubin levels. The AEs were asymptomatic and reversible with either temporary drug discontinuation or administration of glucocorticoids. A similar study terminated because of serious AEs was the combination of vemurafenib, ipilimumab, and DNE3 (a PI3K–AKT inhibitor) in advanced melanoma patients (NCT02095652). In order to analyze the underlying mechanisms of targeted therapy-related toxicities, an observational study is being conducted in patients with advanced solid tumors treated with standard palliative targeted therapies (NCT01758575). Monotherapies include antiangiogenic RTK inhibitors, EGFR inhibitors, mTOR inhibitors, BRAF-inhibitors, and ipilimumab. The study will investigate toxicity both at the systemic and local tissue level.

In spite of these reported toxicities, protocols designed with other drug combinations are currently being conducted. A phase II study assessing a sequential design that combines vemurafenib followed by ipilimumab has completed patient recruitment and results are expected for 2015 (NCT01673854). A new phase I/II trial studying concurrent treatment with dabrafenib and ipilimumab is ongoing (NCT02200562). Another phase I trial is assessing the co-administration of the BMS-908662 pan-RAF inhibitor and ipilimumab (NCT01245556). A combination of ipilimumab, dabrafenib, and trametinib is being assessed in advanced BRAF^V600^ CM patients. One such study is a four-arm, randomized trial with a sequential design that initiated treatment with dabrafenib and/or trametinib followed by ipilimumab (NCT01940809). Yet another open trial is testing a concomitant schedule; a doublet arm in which patients receive a running cycle of dabrafenib followed by co-administration with ipilimumab, and a triplet arm where patients receive dabrafenib, trametinib, and ipilimumab (NCT01767454).

An interesting option for c-KIT mutated melanoma and GIST patients is a dose escalation schedule with cycles of imatinib followed by ipilimumab, currently underway (NCT01738139). Regarding the PD-1–PD-L1 axis, pembrolizumab plus dabrafenib and Trametinib concomitant administration is being assessed in advanced melanoma patients. This study replaces pembolizumab with a placebo and conducts dabrafenib and trametinib monotherapy arms (NCT02130466). When nivolumab and ipilimumab are co-administered there are discernable induction and maintenance phases. Furthermore, MEDI4736 (PD-L1) in combination with oncogene-targeted therapies is being studied in advanced patients (NCT02027961). Patients with BRAF^V600E/K^ tumors will receive dabrafenib, trametinib, and MEDI4736 combination therapy. Patients with BRAF mutation-negative tumors will only receive trametinib plus MEDI4736. A randomized trial in BRAF^V600^ stage III–IV melanoma patients is being conducted with multi-therapy combination (NCT02224781). The trial compares a group treated with dabrafenib plus trametinib followed by ipilimumab plus nivolumab in the case of progression, to a group treated with ipilimumab plus nivolumab followed by dabrafenib plus trametinib at progression. The primary outcome focuses on OS; the secondary outcome on PFS and toxicities. Additional outcomes include genetic characteristics and symptom burden.

### Vaccines and oncogene-targeted therapies

Besides inhibiting target kinases in cancer cells, dasatinib also inhibits a wide variety of kinases, such as src, tec, syk, and gck-families, which are essential for immune system function (79). Chronic myeloid leukemia patients have an increased proportion of granzyme B-expressing T cells at diagnosis, which is further increased by dasatinib therapy. Furthermore, dasatinib-treated CML patients have a higher proportion of effector CD4^+^ T cells that differentiate into Th1-type cytokine producers and are capable of producing IFN-γ, which is key for tumor control (80). Interestingly, although reports have indicated that dasatinib inhibits T cell activation via a blockade of lymphocyte-specific protein tyrosine kinase (Lck)-mediated proximal T cell receptor signaling *in vitro* (81), when administered *in vivo* dasatinib can profoundly enhance T effector cell activation, expansion, and function (82). In preclinical models, Yang *et al*. have recently reported that dasatinib treatment in BALB/c mice bearing P815 mastocytomas improved the recruitment of T cells into the tumor and surrounding stroma, an immunological response required to achieve the clinical benefits associated with therapeutic vaccines (83). Thus, a combinatorial protocol was tested in mice bearing established sub-cutaneous M05 (B16.OVA) melanoma. This protocol used specific vaccination with an OVA-peptide loaded DC-vaccine to activate and expand tumoricidal CD8^+^ T cells plus systemic administration of dasatinib to facilitate the refined targeting of vaccine-induced effector T cells to the tumor microenvironment. This approach provided a superior anti-tumor response to either single-agent modality alone. The increased efficacy of the combinatorial treatment included a reduction in the hypoxic-signaling associated with reduced levels of immunosuppressive CD11b^+^Gr1^+^ myeloid-derived suppressor cells and Treg populations in the melanoma microenvironment. Furthermore, dasatinib in combination with the DC-vaccine upregulated Type-1 T cell-recruiting CXCR3-ligand chemokines in the tumor stroma, which correlated with the activation and recruitment of Type-1, vaccine-induced CXCR3^+^CD8^+^ TILs and CD11c^+^ DC into the tumor. This combinatorial approach resulted in a profound “spreading” of the repertoire of melanoma-associated Ags recognized by CD8^+^ TILs (82). This evidence provided the rationale for a phase I clinical study of dasatinib in combination with a DC-cell based vaccine loaded with tumor blood vessel Ag (TBVA) in metastatic melanoma patients, which is currently ongoing (NCT01876212).

A newly discovered tumor cell escape mechanism is through the upregulation of the tryptophan-catabolizing enzyme known as IDO. Tryptophan is essential for the function of T cells; hence, the depletion of Trp leads to T cell anergy and apoptosis (84). IDO upregulation leads to cancer progression by suppressing T cell immunity, thereby elucidating IDO as a novel potential target for anticancer therapy. The IDO pathway is linked to Treg biology, since IDO-expressing DCs induce the differentiation of naïve CD4^+^ cells toward a FoxP3^+^ phenotype (84). Cancer patients possess spontaneous IDO-peptide specific T cells that are able to recognize and kill both IDO positive tumor cells and DCs (85). Targeting IDO is being explored in clinical trials for patients with metastatic solid tumors using IDO inhibitors such as DMT-1 and INCB024360 or IDO-peptide vaccines. The vaccine combinatorial approach may induce long-lasting IDO-specific memory T cells that could re-activate and be recruited to the tumor site when needed. Targeting IDO by a synthetic peptide vaccine [IDOlong; IDO (194–214) peptide sequence: DTLLKALLEIASCLEKALQVF] in combination with ipilimumab or vemurafenib was tested in a recently completed phase I clinical study for metastatic melanoma patients (NCT02077114). The study results have not yet been published, but will evaluate safety and tolerability as well as immune response. Reactivity to epitopes nested within the sequence of the peptide used for vaccination will be assessed in T cells from peripheral blood at different times. Reactivity will be assessed using ELISPOT (IFN-γ and TNF-α) and fluorochrome-conjugated HLA tetramers to enumerate the frequency of CD8^+^ T cell specific precursors for epitopes within the peptide sequence.

### Other immunotherapies and oncogene-targeted therapies

IL-2 is an immunotherapy drug that increases the proliferation of the T lymphocytes and NK-cells responsible for targeting and eliminating cancer cells. Unfortunately, this elimination is accompanied by high toxicity, since IL-2 is used in high-dose regimen. As previously mentioned, targeting of mutated BRAF^V600E^ with vemurafenib increases Ag expression on the surface of melanoma cells, which can make them easier targets for cytotoxic T lymphocytes and NK-cells. This suggests that combining BRAF-targeted therapy with IL-2 could contribute to increased tumor destruction. This hypothesis is being tested in a phase II clinical study (NCT01754376). The study design consists in primary treatment with oral vemurafenib BID for 2 weeks followed by one course of aldesleukin intravenous infusion (12 weeks long). Patients will also receive vemurafenib twice daily during the course of aldesleukin. Since this is a single arm study the efficacy of the vemurafenib/aldesleukin combination, measured by PFS, will be evaluated in comparison to an historic control of vemurafenib alone. The study will assess response rate, OS, safety, and toxicity. Preclinical data indicating that pharmacologic inhibition of mutated BRAF enhance the immunogenicity of melanoma without adversely affecting the cellular immune response will also be assessed. The study will explore biomarkers that may be relevant to predicting patient responsiveness to vemurafenib and aldesleukin, explaining primary or acquired resistance to vemurafenib, indicating the pharmacodynamic effects of vemurafenib and monitoring the disease. Another open-label, uncontrolled, two-arm, multi-center study has been designed to assess CR rate in BRAF^V600^ metastatic melanoma patients who have received vemurafenib plus HD IL-2 (NCT01683188). Patients will initially receive treatment with vemurafenib interspersed with two courses of HD IL-2. Eligible patients have been on vemurafenib therapy for 0–18 weeks, those patients previously taking vemurafenib must have responding or stable disease and all must meet the requirements for HD IL-2 dosing. Two cohorts will be enrolled, differing only in how they are characterized prior to HD IL-2 treatment: Cohort 1, patients naïve to vemurafenib and HD IL-2 therapy; Cohort 2, patients who have been on vemurafenib therapy for >7–18 weeks with stable or responding disease before starting HD IL-2. Patients in both cohorts will discontinue vemurafenib prior to each treatment with HD IL-2 and resume dosing after each discharge. Patients will receive up to two courses (four cycles) of HD IL-2.

IFN-α acts via engagement of a Type I IFN receptor consisting of two subunits (IFNAR1 and IFNAR2) followed by signaling through the JAK and the STAT family to activate the transcription of IFN-stimulated genes [reviewed in Ref. (58)]. A phase I/II trial has been launched to test this combination in advanced melanoma patients. This study is designed to evaluate the safety and the efficacy of the vemurafenib/PEG–interferon combination as well as the IFNAR1 upregulation induced by this treatment. Previous preclinical work reported by Kumar *et al*. demonstrated in a BRAF^V600E^ melanoma cell line that oncogenic BRAF–MAPK signaling leads to the acceleration of IFNAR1 degradation by inducing βTrcp2 expression (86). Pharmacologic inhibition of either RAF or MEK1 stabilized IFNAR1 in melanoma cells and decreased their tumorigenicity. The destabilization and downregulation of IFNAR1 in BRAF^V600E^ melanomas might account for the suboptimal anti-proliferative effects of IFN. Preclinical results obtained in murine models suggest that a combination treatment of IFN-α with RAF or MAPK inhibitors could decrease BRAF signaling and βTrcp2 expression and should prevent the rapid degradation of IFNAR1. This increase in the extent of IFN-α signaling could provide a promising strategy for treatment of melanoma. However, *in vitro* results also suggests that the BRAF-inhibitor BAY 43-9006 (Sorafenib) may decrease the extent of direct anti-proliferative responses to IFN-α by directly inhibiting the catalytic activity of JAK (86).

As previously mentioned, another immunotherapeutic strategy for the treatment of metastatic melanoma is ACT, with autologous TIL infusion after a lymphodepleting regimen, combined with IL-2 administration to support TIL growth. Furthermore, PLX-4720 significantly increased tumor infiltration by adoptively transferred T cells *in vivo* and enhanced the anti-tumor activity of ACT in a xenograft model with labeled T cells and human CM cell lines (87). This increase in tumor infiltration was primarily an effect of the inhibition of VEGF secretion by tumor cells. Currently, two open clinical studies are testing the combination of vemurafenib with ACT patients in BRAF^V600^ melanoma. In the study NCT01585415, the safety of vemurafenib administration will be evaluated both before and concurrent to autologous TIL infusion along with high HD-IL2, following a non-myeloablative lymphodepleting preparative regimen with Fluradabine. Furthermore, this study may provide information on how this combination of therapies mediates clinical tumor regression in patients, as well as the immunologic impact of BRAF^V600E^ inhibition on the lymphoid infiltrate in melanoma deposits. In another study, the efficacy and side effects of this combination will also be analyzed (NCT01659151). This combination of therapies is designed to take advantage of the vemurafenib-induced upregulation of target Ags before TIL infusion. Furthermore, BRAF inhibition could contribute to the “autovaccination” of patients as tumor destruction leads to epitope exposition and immune effector elicitation. However, some questions will remain unanswered, since treatment with vemurafenib before harvesting the tumor for use in TIL could contribute to an increase in T cell infiltrate in the melanoma lesions. This could enrich TIL samples and potentially improve ACT outcome. These studies may elucidate whether the pre-ACT lymphodepleting regimen, necessary for ACT success, impairs vemurafenib treatment. Knight *et al*. have suggested that this may be the case if vemurafenib anti-tumor activity is mediated by resident immune cells (71). Thus, further research is necessary to assess the timing of TIL recovery, the lymphodepletion regimen and vemurafenib administration.

## Future Directions and Perspectives

Combination therapies comprised of oncogene-targeted and immunotherapeutic strategies are a promising, emerging approach to melanoma treatment. Oncogenes impact not only tumor proliferation, but also other cancer hallmarks such as immune system evasion, which favors a tumor-permissive immune microenvironment. There is increasing evidence that oncogene-targeted inhibitors not only induce tumor cell death, but can also contribute to the restoration of an anti-tumor immune microenvironment. Oncogene-targeted therapies offer the possibility of rapid bulky tumor elimination, generate a vaccine-like boost and Ag tumor spreading and favor tumor infiltration by immune effectors, all of which may contribute to more extensive and durable tumor control.

Disease stage is a significant consideration in the design of combination strategies. In advanced metastatic melanoma, response kinetics has been a key factor in agent selection. Patients who have rapidly progressing tumors with druggable mutations can benefit from oncogene-targeted drugs first, allowing the elimination of important tumor masses in a short time. Sequential immunotherapy administration could cause tumor immune infiltration and therefore, promote durable immune control of disease dissemination. Concomitant administration of oncogene-targeted therapies with immunotherapy has produced serious AEs, though results are pending from additional clinical studies. Advanced patients with stable or slowly progressing disease might benefit from immunotherapies that act gradually but achieve long-lasting responses. Recent results with the antagonist mAb pembrolizumab are encouraging for patients who move on to immunotherapy or oncogene-targeted therapies. Instead, in the adjuvant setting, cancer vaccines could be the first choice to elicit a robust immune response. Their combination with an immune checkpoint blockade could enhance and prolong immune stimulation and effector numbers.

The choice of the best therapeutic design for combining immunotherapy with oncogene-targeted drugs (sequential vs. concurrent schemes), avoidance of toxicity and induction of long-lasting clinical responses are controversial and challenging issues. There is an urgent need for biomarker development through histologic and genomic analysis of the tumor and its microenvironment. Biomarkers will be able to predict which patients are likely to have a clinical benefit and which will have severe AEs. The use of biomarkers will give rise to a more personalized medicine for cancer treatment.

The development of more effective melanoma treatments based on oncogene-targeted therapy in combination with immunotherapy can emerge from novel hypothesis-testing and biomarker-driven clinical trials designed to optimize both drug dosing and immunotherapy scheduling. Ongoing and future clinical studies will certainly contribute to this future and illuminate these new roads for melanoma therapy.

## Conflict of Interest Statement

The authors declare that the research was conducted in the absence of any commercial or financial relationships that could be construed as a potential conflict of interest.

## Abbreviations

ACT, adoptive cell therapy; AE, adverse events; Ag, antigen/s; APC, antigen-presenting cells; CM, cutaneous melanoma; CR, complete response; DC, dendritic cells; DFS, disease-free survival; EMEA, European Medicines Agency; FDA, Federal Drug Administration; HD IL-2, high-dose interleukin-2; IFA, incomplete Freund’s adjuvant; irAEs, immune-related adverse events; mAbs, monoclonal antibodies; ORR, overall response rate; OS, overall survival; PFS, progression-free survival; RTK, receptor tyrosine kinase; TIL, tumor infiltrating lymphocytes.
